# Predicting 3-year all-cause mortality in rectal cancer patients based on body composition and machine learning

**DOI:** 10.3389/fnut.2025.1473952

**Published:** 2025-03-03

**Authors:** Xiangyong Li, Zeyang Zhou, Xiaoyang Zhang, Xinmeng Cheng, Chungen Xing, Yong Wu

**Affiliations:** Department of Gastrointestinal Surgery, The Second Affiliated Hospital of Soochow University, Suzhou, China

**Keywords:** rectal cancer, nutrition, prognosis, machine learning, predictive model

## Abstract

**Objectives:**

The composition of abdominal adipose tissue and muscle mass has been strongly correlated with the prognosis of rectal cancer. This study aimed to develop and validate a machine learning (ML) predictive model for 3-year all-cause mortality after laparoscopic total mesorectal excision (LaTME).

**Methods:**

Patients who underwent LaTME surgery between January 2018 and December 2020 were included and randomly divided into training and validation cohorts. Preoperative computed tomography (CT) image parameters and clinical characteristics were collected to establish seven ML models for predicting 3-year survival post-LaTME. The optimal model was determined based on the area under the receiver operating characteristic curve (AUROC). The SHAPley Additive exPlanations (SHAP) values were utilized to interpret the optimal model.

**Results:**

A total of 186 patients were recruited and divided into a training cohort (70%, *n* = 131) and a validation cohort (30%, *n* = 55). In the training cohort, the AUROCs of the seven ML models ranged from 0.894 to 0.949. In the validation cohort, the AUROCs ranged from 0.727 to 0.911, with the XGBoost model demonstrating the best predictive performance: AUROC = 0.911. SHAP values revealed that subcutaneous adipose tissue index (SAI), visceral adipose tissue index (VAI), skeletal muscle density (SMD), visceral-to-subcutaneous adipose tissue ratio (VSR), and subcutaneous adipose tissue density (SAD) were the five most important variables influencing all-cause mortality post-LaTME.

**Conclusion:**

By integrating body composition, multiple ML predictive models were developed and validated for predicting all-cause mortality after rectal cancer surgery, with the XGBoost model exhibiting the best performance.

## 1 Introduction

According to statistics, colorectal cancer ranks third among malignancies in terms of incidence and is the second leading cause of cancer-related deaths, with rectal cancer specifically occupying the eighth position and accounting for one-third of all colorectal malignancy cases (1, 2). Importantly, survival rates for patients diagnosed at early to intermediate stages decline with advancing tumor stages (3), underscoring the critical need for accurate prognosis prediction in this patient population.

The classical Tumor-Node-Metastasis (TNM) staging system is the primary basis for evaluating prognosis and guiding treatment strategies in rectal cancer patients (4). However, this system’s precision and reliability remain insufficient to fully meet the comprehensive demands of clinical practice (5, 6). Consequently, there is a pressing need to explore and incorporate multidimensional biomarkers and clinical indicators to optimize further and refine the prognostic prediction framework for rectal cancer. Research has demonstrated that the content and proportions of visceral adipose tissue, subcutaneous adipose tissue, and skeletal muscle are correlated with clinical outcomes in colorectal cancer patients, exerting substantial influences on disease onset, progression, and prognosis (7–9). These tissue areas and densities can be conveniently and accurately obtained through preoperative CT/MR imaging modalities (10, 11).

In recent years, machine learning (ML), a novel form of artificial intelligence (AI), has gained increasing prominence in data mining and has been widely applied in medical data analysis due to its prowess in handling large datasets (12, 13). Prior studies have predominantly focused on the relationship between individual or multiple abdominal components and rectal cancer prognosis (9–11), as well as the development and validation of predictive models such as nomograms (14, 15). Nevertheless, there is a paucity of reports regarding the integration of abdominal adipose tissue and muscle with ML models to predict postoperative outcomes following LaTME.

Thus, we aim to develop a model that predicts 3-year all-cause mortality in patients after LaTME. This model has the potential to facilitate the early identification of patients with shorter survival prognoses, enabling timely interventions for optimal survival outcomes. Furthermore, we aspire for this model to guide the formulation of standardized nutritional protocols and the refinement of nutritional therapies.

## 2 Materials and methods

### 2.1 Patients and study design

This study enrolled 186 patients who underwent LaTME from January 2018 to December 2020 in the Gastrointestinal Surgery Department of the Second Affiliated Hospital of Soochow University. Inclusion criteria were: (1) preoperative pathological diagnosis confirming rectal cancer; (2) complete CT scans and clinical data within 2 weeks prior to surgery; and (3) surgical approach being LaTME.

**Figure 1:**
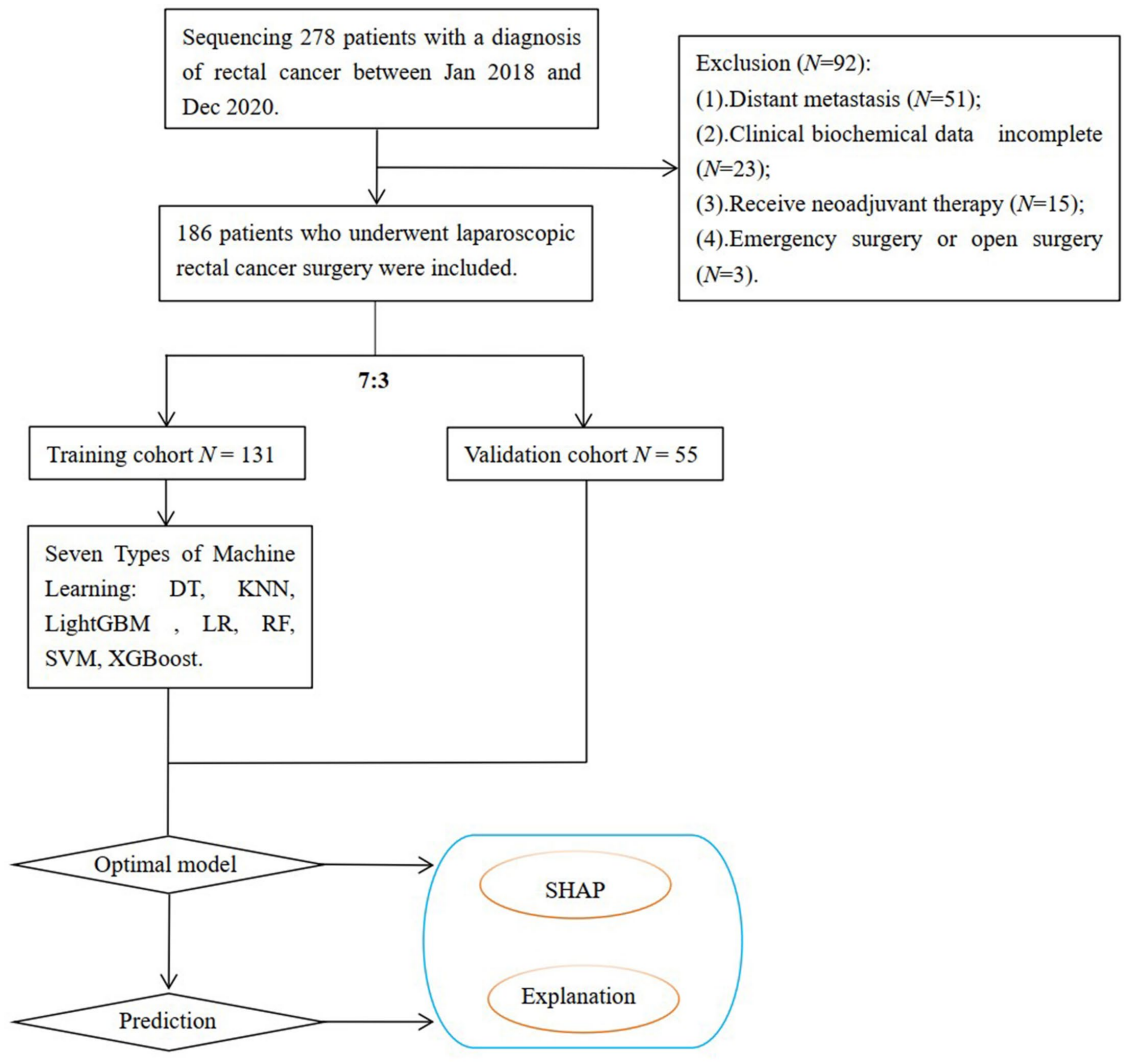
Flowchart of the study.

Exclusion criteria were: (1) emergency surgery; (2) open surgery; (3) preoperative adjuvant therapies such as radiotherapy or chemotherapy; and (4) clinical stage IV or inoperable cases due to massive tumors (Figure 1). All procedures performed in this study adhered to the principles outlined in the Declaration of Helsinki of 1964. Studies involving human subjects were reviewed and approved by the Ethics Committee of the Second Affiliated Hospital of Soochow University (NO: JD-HG-2024-037).

### 2.2 Data collection

**Figure 2:**
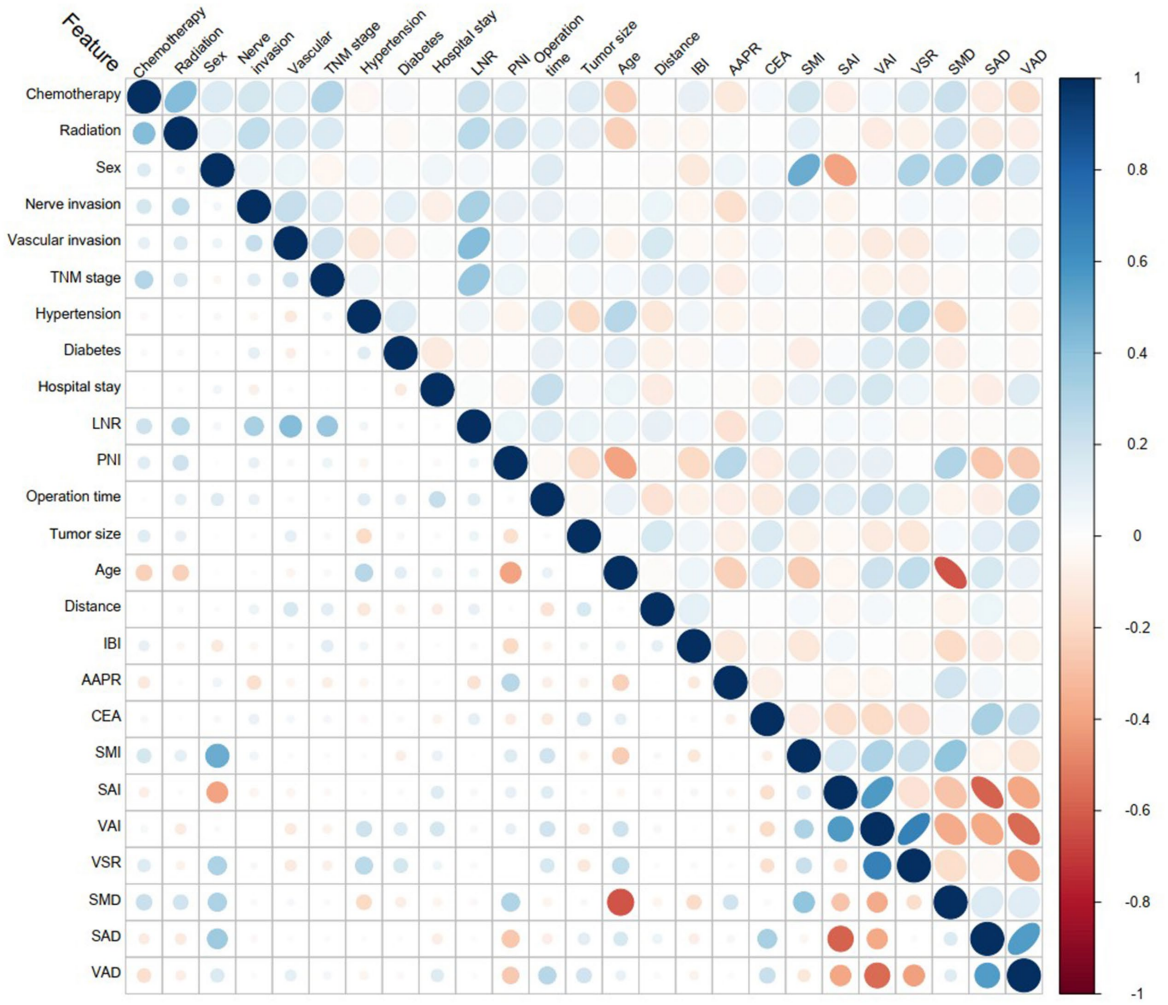
Variable correlation heatmap. The blue color represents positive correlation, while red indicates negative correlation. The intensity of the color signifies the strength of the correlation.

For the patients included in this study, the following indicators were retrospectively collected from our hospital’s electronic medical record system:

1. Baseline Characteristics of the Patients: Age, gender, comorbidities (hypertension, diabetes), postoperative adjuvant therapy, operative duration, and postoperative hospital stay.
2. CT Measurement Parameters: Skeletal muscle index (SMI), subcutaneous adipose tissue index (SAI), visceral adipose tissue index (VAI), skeletal muscle density (SMD), subcutaneous adipose tissue density (SAD), visceral adipose tissue density (VAD), and visceral-to-subcutaneous adipose tissue area ratio (VSR).
3. Blood Laboratory Indicators: Albumin-to-alkaline phosphatase ratio (AAPR) (16), inflammatory burden index (IBI) (17), prognostic nutritional index (PNI) (18), and carcinoembryonic antigen (CEA).
4. Pathological Characteristics: TNM staging, nerve invasion, vascular invasion, and lymph node positivity ratio (LNR). No significant correlations were observed among these variables (Figure 2).

### 2.3 Image analysis

For the analysis of body composition, Slice-O-Matic software (V5.0; TomoVision) was employed to calculate visceral adipose tissue (VAT) and subcutaneous adipose tissue (SAT) parameters at the L4-L5 intervertebral disc level, using two consecutive CT transverse slices (5 mm apart) and averaging the results. Anatomical knowledge and tissue-specific Hounsfield unit (HU) ranges were applied to delineate cross-sectional areas, with skeletal muscle ranging from −29 to +150 HU, VAT from −150 to −50 HU, and SAT from −190 to −30 HU (10). Additionally, the mean radiodensity of each tissue was obtained. For each patient’s CT, two individuals (Li and Zhou), trained in the software’s usage, independently outlined the target regions, and the average of their measurements was taken. In cases of significant discrepancy, a third party verified the outlines (Supplementary Figure S1).

The SMI, SAI, and VAI were derived using normalized areas of skeletal muscle, SAT, and VAT, respectively. Skeletal muscle tissue density (SMD), subcutaneous adipose tissue density (SAD), and visceral adipose tissue density (VAD) were obtained by averaging the radiodensity across the total cross-sectional area based on preoperative CT scans. The visceral-to-subcutaneous fat area ratio (VSR) was calculated to assess visceral obesity (19).

### 2.4 Follow-up visits

This study employed a combined approach of outpatient revisit and telephone follow-up. The initial follow-up was conducted 1 month post-surgery, followed by visits every 1–3 months during the first postoperative year, transitioning to every 6 months in the second year. From the third year onwards, annual follow-ups were scheduled. The follow-up period concluded in December 2023 or upon the patient’s demise, with the primary outcome measure being survival outcome (alive or dead) at the 3-year post-operative mark.

### 2.5 Establishment of ML model

Patients were randomly assigned in a 7:3 ratio to a training cohort (*n* = 131) and a validation cohort (*n* = 55). We leveraged ML models, including Decision Tree (DT), K-Nearest Neighbors (KNN), Light Gradient Boosting Machine (LightGBM), Logistic Regression (LR), Random Forest (RF), Support Vector Machine (SVM), and Extreme Gradient Boosting (XGBoost), to construct and validate our models. Grid search, coupled with 5-fold cross-validation, was employed to identify optimal parameters for each of the seven ML models, optimizing for the area under the receiver operating characteristic curve (AUROC). We calculated sensitivity, specificity, accuracy, positive predictive value (PPV), negative predictive value (NPV), recall, and F-score to comprehensively evaluate model performance.

In comparing the predictive performance of these ML models, we relied on AUROC as well as a composite assessment of multiple metrics to gauge the overall efficacy. Regarding model interpretability, we utilized Shapley Additive exPlanations (SHAP) values to elucidate the clarity and explainability of the best-performing model. Specifically, we generated SHAP beeswarm plots ranking the predictive variables based on the optimal model selected, thereby facilitating insight into their relative importance. In addition, we elucidate the effect of individual attributes on the predictive power of the optimal model, thus providing a localized explanation.

### 2.6 Statistical methods

All statistical analyses and visualizations in this study were conducted using the R programming language (version 4.4.1). The normality of continuous variables was assessed using the Shapiro–Wilk test and Q-Q plots. Descriptive statistics for normally distributed variables are presented as mean ± standard deviation [mean (SD)], whereas median and interquartile range (median [IQR]) are reported for non-normally distributed variables. Categorical variables are expressed as absolute numbers and percentages [*n* (%)]. The Student’s t-test was employed for comparisons of continuous variables, and the chi-squared (*χ*^2^) test was used for categorical variables. Statistical significance was set at a two-sided *p*-value of <0.05.

## 3 Results

### 3.1 Baseline characteristics

A total of 186 patients were recruited for this study, comprising 112 males (60.22%) and 74 females (39.78%). The median age was 66 years. At the 3-year postoperative follow-up, 146 patients were alive, while 40 had deceased. Among the participants, 76 had comorbid hypertension, and 21 had diabetes. Supplementary Table S1 summarizes the baseline characteristics comparison between different survival outcomes. Notably, no significant differences were observed in clinical features and CT parameters between the training cohort and the validation cohort (*p* > 0.05) (Table 1).

**Table 1:** Baseline patient characteristics.

Variables	Total (*n* = 186)	Training cohort (*n* = 130)	Validation cohort (*n* = 56)	*p*-value
Baseline characteristics of the patients				
Age [mean (SD), year]	66.05 ± 11.12	67.22 ± 11.02	63.32 ± 10.95	0.028
Sex, *n* (%)				0.927
Female	74 (39.78)	52 (40.00)	22 (39.29)	
Male	112 (60.22)	78 (60.00)	34 (60.71)	
Hypertension, *n* (%)				0.131
No	104 (55.91)	68 (52.31)	36 (64.29)	
Yes	82 (44.09)	62 (47.69)	20 (35.71)	
Diabetes, *n* (%)				0.241
No	165 (88.71)	113 (86.92)	52 (92.86)	
Yes	21 (11.29)	17 (13.08)	4 (7.14)	
Chemotherapy, *n* (%)				0.951
No	87 (46.77)	61 (46.92)	26 (46.43)	
Yes	99 (53.23)	69 (53.08)	30 (53.57)	
Radiation, *n* (%)				0.265
No	154 (82.80)	105 (80.77)	49 (87.50)	
Yes	32 (17.20)	25 (19.23)	7 (12.50)	
Operation time [median (IQR), min]	225.00 (195.00, 280.00)	227.50 (195.00, 280.00)	220.00 (187.50, 276.25)	0.522
Postoperative hospital stay [median (IQR), day]	11.00 (9.00, 13.00)	11.00 (9.00, 13.75)	10.00 (9.00, 12.00)	0.461
CT measurement parameters				
Tumor size [median (IQR), mm]	35.00 (30.00, 50.00)	35.00 (25.75, 50.00)	40.00 (30.00, 51.25)	0.139
Distance from the tumor to anus [median (IQR), cm]	9.55 (5.43, 11.10)	9.95 (5.43, 11.10)	8.90 (5.47, 10.85)	0.504
SMI [mean (SD), cm/kg^2^]	43.66 ± 8.10	43.59 ± 8.06	43.82 ± 8.25	0.854
SAI [mean (SD), cm/kg^2^]	47.76 ± 21.76	46.39 ± 20.31	50.94 ± 24.71	0.192
VAI [mean (SD), cm/kg^2^]	40.72 ± 22.23	40.59 ± 21.82	41.04 ± 23.34	0.898
SMD [mean (SD), U]	32.67 ± 7.17	32.50 ± 7.30	33.07 ± 6.93	0.617
SAD [mean (SD), U]	−97.34 ± 9.03	−97.33 ± 8.82	−97.36 ± 9.61	0.986
VAD [mean (SD), U]	−94.08 ± 8.36	−94.12 ± 8.36	−93.98 ± 8.43	0.917
VSR [median (IQR)]	0.79 (0.56, 1.13)	0.86 (0.59, 1.15)	0.73 (0.52, 1.01)	0.174
Blood laboratory indicators				
AAPR [mean (SD)]	0.54 ± 0.16	0.54 ± 0.17	0.54 ± 0.13	0.971
IBI [median (IQR)]	12.10 (9.43, 17.50)	11.93 (9.47, 17.05)	12.49 (9.17, 18.18)	0.944
PNI [median (IQR)]	49.90 (46.23, 52.38)	49.90 (46.05, 52.60)	50.00 (47.10, 52.08)	0.97
CEA [median (IQR), ng/L]	3.70 (2.51, 6.54)	3.63 (2.52, 5.86)	3.77 (2.52, 9.88)	0.404
Pathological characteristics				
TNM stage, *n* (%)				0.059
I/II	128 (68.82)	84 (64.62)	44 (78.57)	
III	58 (31.18)	46 (35.38)	12 (21.43)	
Nerve invasion, *n* (%)				0.685
No	146 (78.49)	101 (77.69)	45 (80.36)	
Yes	40 (21.51)	29 (22.31)	11 (19.64)	
Vascular invasion, *n* (%)				0.08
No	152 (81.72)	102 (78.46)	50 (89.29)	
Yes	34 (18.28)	28 (21.54)	6 (10.71)	
LNR [median (IQR)]	0.00 (0.00, 0.13)	0.00 (0.00, 0.16)	0.00 (0.00, 0.06)	0.056

TNM, Tumor-Node-Metastasis [The 8th edition of the American Joint Committee on Cancer (AJCC) staging system]; SMI, skeletal muscle index; SAI, subcutaneous adipose tissue index; VAI, visceral adipose tissue index; SMD, skeletal muscle density; SAD, subcutaneous adipose tissue density; VAD, visceral adipose tissue density; VSR, visceral-to-subcutaneous adipose tissue area ratio; AAPR, albumin-to-alkaline phosphatase ratio; IBI, inflammatory burden index; PNI, prognostic nutritional index; CEA, carcinoembryonic antigen.

### 3.2 Establishment and evaluation of the model

**Figure 3:**
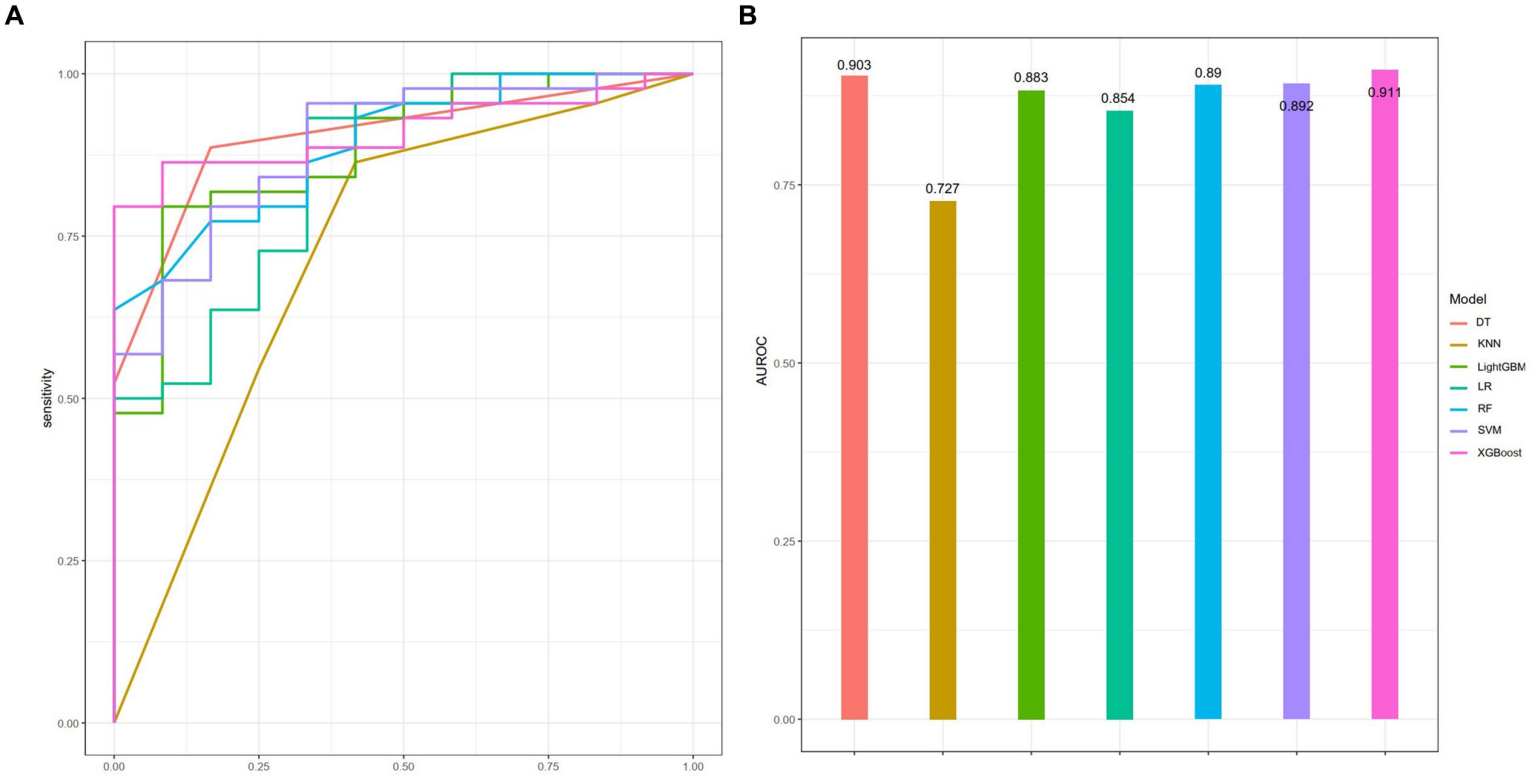
Performance of seven ML models in the training cohort as assessed by the AUROC **(A)**, with corresponding visual representation in a bar chart **(B)**.

A total of 186 patients were enrolled and randomly assigned to a training cohort (*n* = 131) and a validation cohort (*n* = 55) in a 7:3 ratio. Seven machine learning models were selected for this study: DT, KNN, LightGBM, LR, RF, SVM, and XGBoost. Among these, LR exhibited the optimal performance in the training cohort, with an AUROC of 0.949 (Supplementary Table S2). Conversely, in the validation cohort, XGBoost surpassed the others, achieving an AUROC of 0.911 (Figure 3).

To assess the predictive efficacies of these seven models, we employed the DeLong test, which revealed that only the KNN model displayed a statistically significant difference in predictive performance compared to the rest (*p* < 0.05). The calibration curve and DCA for each model are presented in Supplementary Figures S2, S3, respectively. All other models, without notable variations among themselves, demonstrated superior predictive capabilities (Supplementary Table S3). Considering the AUROC, sensitivity, and other pertinent metrics in the validation set (Table 2), XGBoost stood out as the most performant mode. Consequently, XGBoost was chosen for further predictive analysis and exploration.

**Table 2:** Evaluate the predictive performance of seven ML models in the validation cohort.

Indicator	Models						
	XGBoost	DT	SVM	RF	LightGBM	LR	KNN
AUROC	0.911	0.903	0.892	0.89	0.883	0.854	0.727
Accuracy	0.804	0.875	0.804	0.768	0.804	0.839	0.589
Sensitivity	0.750	0.833	0.841	0.795	0.818	0.886	0.545
Specificity	1	0.886	0.667	0.667	0.75	0.667	0.75
PPV	1	0.667	0.902	0.897	0.923	0.907	0.889
NPV	0.522	0.951	0.533	0.471	0.529	0.615	0.31
Balance accuracy	0.875	0.86	0.754	0.731	0.784	0.777	0.648
Precision	1.000	0.667	0.902	0.897	0.923	0.907	0.889
Recall	0.750	0.667	0.841	0.795	0.818	0.886	0.545
F-score	0.857	0.857	0.871	0.843	0.867	0.897	0.676

DT, Decision Tree; KNN, K-Nearest Neighbors; LightGBM, Light Gradient Boosting Machine; LR, Logistic Regression; RF, Random Forest; SVM, Support Vector Machine; XGBoost, Extreme Gradient Boosting; PPV, Positive Predictive Value; NPV, Negative Predictive Value.

### 3.3 Model explainability

**Figure 4:**
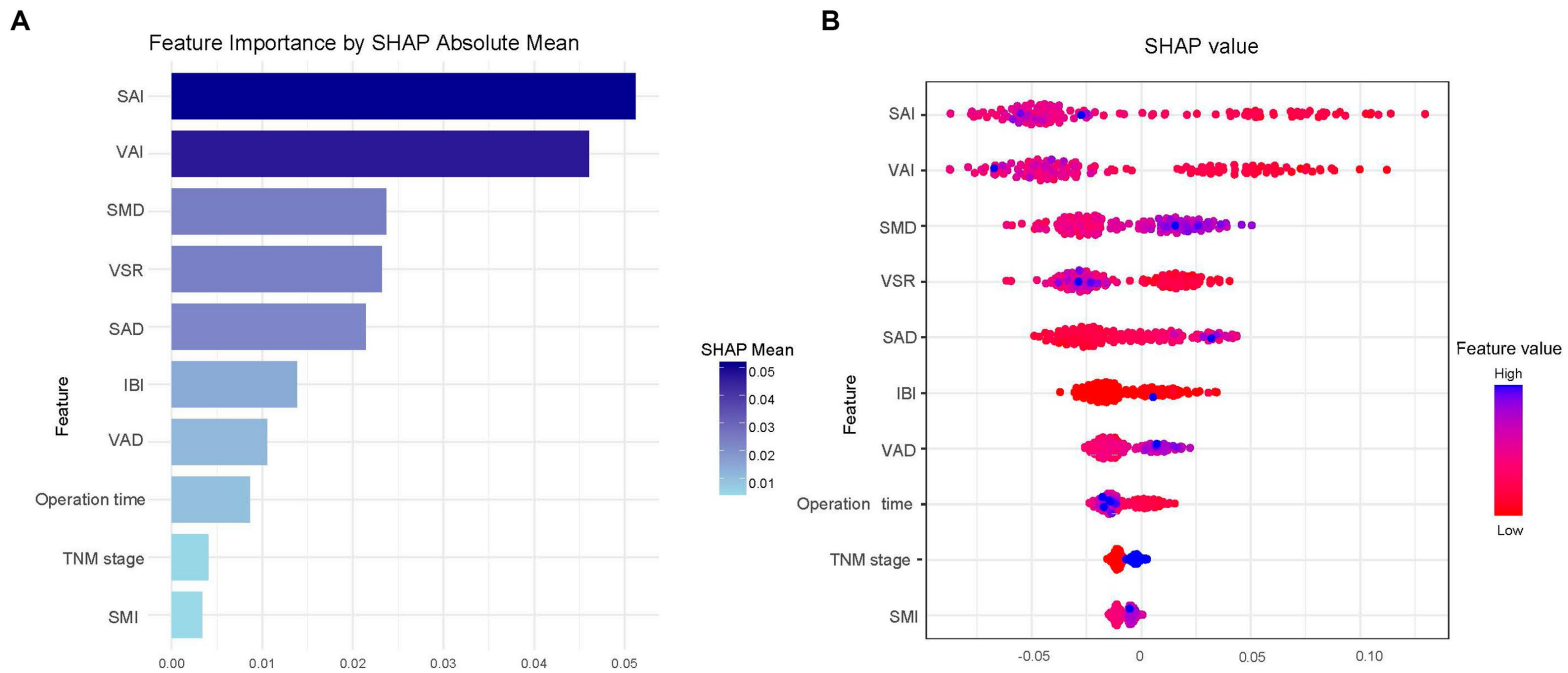
SHAP summary plot for the top 10 clinical features contributing to the XGBoost model. SHAP feature importance is measured as the mean absolute Shapley values **(A)** and the attributes of the features in the model **(B)**.

**Figure 5:**
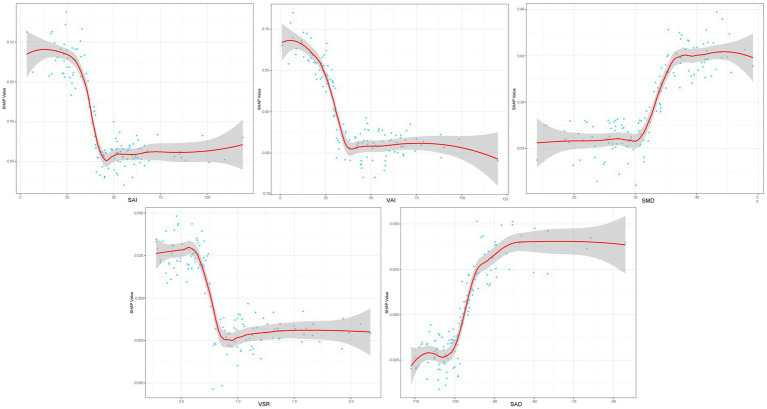
SHAP dependency plot for the top 5 clinical features contributing to the XGBoost model.

As XGBoost emerged as the optimal model for predicting all-cause mortality in rectal cancer, Figure 4A presents the ranking of feature importance within the XGBoost model. The top five most influential features of the XGBoost model are SAI, VAI, SMD, VSR, and SAD. Furthermore, Figure 4B depicts how individual features contribute to the predictive effect on the dependent variable within the model, with each point representing the SHAP value of a specific feature for a sample. Figure 5 illustrates the trends in how these five features impact the model’s predictions.

## 4 Discussion

This study established and validated seven ML models to predict 3-year all-cause mortality following LaTME. Among the compared ML models, XGBoost demonstrated notable superiority. To our knowledge, this is the first study utilizing interpretable machine learning based on abdominal body composition to predict 3-year all-cause mortality after LaTME. While various nomogram models have been previously developed (20–22), our work distinguishes itself by introducing a high-performance ensemble machine learning model, which holds the potential to refine strategic resource allocation and inform more effective patient management strategies.

Obesity has been well-established as a risk factor for the development and progression of multiple cancer types, including lung (23), pancreatic (24), gastric (25), and colorectal cancers (26). However, some studies have suggested that obesity may paradoxically serve as a protective factor for certain diseases (27, 28). Nevertheless, the intricate relationship between obesity and prognosis in rectal cancer patients remains largely unelucidated, primarily due to the limitations of BMI, which fails to distinguish between adipose tissue and skeletal muscle or to delineate adipose tissue distribution. One study proposed that quantified fat-free mass index (FFMI) and fat mass-to-fat-free mass ratio (FM/FFM) may better predict functional outcomes in pre-frail elders than BMI (29). Previous research has shown that low skeletal muscle index (SMI) adversely impacts colorectal cancer prognosis (30, 31). Regarding subcutaneous adipose tissue (SAT) and visceral adipose tissue (VAT), most studies have focused on their cross-sectional areas in relation to colorectal cancer prognosis without considering confounding factors such as height. Therefore, quantifying body composition through indices like SAI and VAI can mitigate the influence of confounders, enhancing the accuracy of abdominal obesity assessment. Our study further highlights SMD and SAD as robust predictors of all-cause mortality in rectal cancer patients, consistent with prior findings (10, 32). Feliciano EMC et al. suggested that higher radiological density of VAT or SAT may indicate lower lipid content in adipocytes, potentially reflecting weight loss, a hallmark of disease progression. However, their analysis of patients maintaining stable weight between scans yielded similar results (10), suggesting that the underlying mechanisms may require further investigation.

It is noteworthy that in our study, the SAI emerged as the strongest predictor of three-year survival among rectal cancer patients, aligning with findings from several studies which indicate that a low SAI is independently associated with an increased mortality rate (33, 34). Intriguingly, these studies point out that a low VATI is not an independent risk factor for the prognosis of rectal cancer patients (33, 34). The finding that having a high VAI without concurrent high subcutaneous obesity increases the risk of mortality contrasts with conclusions drawn from multiple studies (35, 36). This discrepancy may be attributed to the influence of demographic factors such as different disease types, age, gender, and ethnicity (37), or it could be related to variations in TNM staging. However, no definitive conclusion has been reached, and further investigation is required.

Our study also highlights SMD and SAD as strong predictors of survival outcomes in rectal cancer patients, consistent with previous research (30, 31). Feliciano et al. suggest that higher VAT or SAT radiodensity may reflect lower lipid content in adipocytes, potentially due to weight loss, which is a hallmark of progressive disease. However, they found similar results when restricting their analysis to patients who maintained stable weight between imaging sessions (31). A clearer mechanism may require further research. In our study, VSR was also identified as a significant predictor, with findings similar to those reported in several other studies (38, 39). Furthermore, VSR is recognized as an effective indicator for assessing body fat distribution. By identifying key variables associated with increased risk, SHAP can facilitate early interventions and personalized treatment planning, enabling more informed and individualized clinical decision-making.

Numerous studies have highlighted the strong association between nutritional-inflammatory indices, such as PNI (40), IBI (41), and AAPR (42), and cancer prognosis. However, in our study, these variables were less influential in the model compared to abdominal muscle and adipose tissue composition. Given the relatively small patient cohort, the contribution of these indices to the model warrants further investigation.

Our study is not without limitations. Firstly, as a single-center retrospective study with a limited patient sample, it cannot fully rule out selection bias. A larger sample size from multiple centers is needed to validate our findings. Increasing the sample size to approximately 3,000 will provide a more robust dataset, enabling more reliable statistical analyses and potentially identifying stronger associations. Secondly, while the study included laboratory tests, clinicopathological features, and abdominal CT parameters, the CT measurements relied on average areas from two planes, which may not fully capture abdominal adipose tissue volume, thereby introducing potential errors. Lastly, due to the limitation of follow-up duration, our study focused solely on the 3-year all-cause mortality rate, without delving into the 5-year or longer-term rectal cancer-specific mortality rate. This, to a certain extent, constrained the accuracy of our model. Consequently, further research in this regard is imperative.

## 5 Conclusion

In summary, we have developed and validated seven machine learning models utilizing CT-derived body composition data to predict 3-year all-cause mortality following LaTME. Notably, the XGBoost model emerged as the most predictive, highlighting SAI, VAI, SMD, VSR, and SAD as the five most significant predictive variables influencing three-year survival post-LaTME. This underscores the potential clinical significance of integrating body composition metrics and advanced machine learning techniques in prognostic assessments for rectal cancer patients undergoing LaTME.

## Data Availability

The original contributions presented in the study are included in the article/Supplementary material, further inquiries can be directed to the corresponding authors.

## Supplementary material

SUPPLEMENTARY TABLE S1 Comparison of baseline data between patients with different survival outcomes.

SUPPLEMENTARY TABLE S2 Evaluate the predictive performance of seven ML models in the training cohort.

SUPPLEMENTARY TABLE S3 Compare the AUROC differences of the seven models using the DeLong test in the training cohort.

SUPPLEMENTARY FIGURE S1 Schematic diagram at the level of the L4/5 spine in the used patient using Slice-O-Matic software.

SUPPLEMENTARY FIGURE S2 The calibration curves for the seven models.

SUPPLEMENTARY FIGURE S3 The clinical impact curves for the seven models.

## Glossary

ML: machine learning
LaTME: laparoscopic total mesorectal excision
CT: computed tomography
AUROC: area under the receiver operating characteristic curve
SHAP: SHAPley Additive exPlanations
TNM: Tumor-Node-Metastasis
SMI: skeletal muscle index
SAI: subcutaneous adipose tissue index
VAI: visceral adipose tissue index
SMD: skeletal muscle density
SAD: subcutaneous adipose tissue density
VAD: visceral adipose tissue density
VSR: visceral-to-subcutaneous adipose tissue area ratio
AAPR: albumin-to-alkaline phosphatase ratio
IBI: inflammatory burden index
PNI: prognostic nutritional index Positive Predictive Value
NPV: Negative Predictive Value
CEA: carcinoembryonic antigen
DT: Decision Tree
KNN: K-Nearest Neighbors
LightGBM: Light Gradient Boosting Machine
LR: Logistic Regression
RF: Random Forest
SVM: Support Vector Machine
XGBoost: Extreme Gradient Boosting
