# Supplementary material for: Predicting 3-year all-cause mortality in rectal cancer patients based on body composition and machine learning
Source: Front Nutr. 2025 Mar 3;12:1473952. doi: 10.3389/fnut.2025.1473952 (PMC11911182; doi:10.3389/fnut.2025.1473952)
Supplement: SUPPLEMENTARY TABLE S1 — Comparison of baseline data between patients with different survival outcomes. [file Data_Sheet_1.docx]

**Supplementary Table S1 Comparison of baseline data between patients with different survival outcomes**

| **Variables** | **Total (n = 186)** | **Alive (n = 146)** | **Death (n = 40)** | ***P*-value** |
| --- | --- | --- | --- | --- |
| **Baseline characteristics of the patients** | | | | |
| Age, (mean [SD], year) | 66.05 ± 11.12 | 66.22 ± 11.26 | 65.42 ± 10.70 | 0.69 |
| Sex, n(%) |  |  |  | 0.288 |
| female | 74 (39.78) | 61 (41.78) | 13 (32.50) |  |
| man | 112 (60.22) | 85 (58.22) | 27 (67.50) |  |
| Hypertension, n(%) |  |  |  | 0.096 |
| No | 104 (55.91) | 77 (52.74) | 27 (67.50) |  |
| Yes | 82 (44.09) | 69 (47.26) | 13 (32.50) |  |
| Diabetes, n(%) |  |  |  | 0.567 |
| No | 165 (88.71) | 128 (87.67) | 37 (92.50) |  |
| Yes | 21 (11.29) | 18 (12.33) | 3 (7.50) |  |
| Chemotherapy, n(%) |  |  |  | 0.8 |
| No | 87 (46.77) | 69 (47.26) | 18 (45.00) |  |
| Yes | 99 (53.23) | 77 (52.74) | 22 (55.00) |  |
| Radiation, n(%) |  |  |  | 0.317 |
| No | 154 (82.80) | 123 (84.25) | 31 (77.50) |  |
| Yes | 32 (17.20) | 23 (15.75) | 9 (22.50) |  |
| Operation time, (median [IQR]), min) | 225.00 (195.00, 280.00) | 227.50 (195.00, 280.00) | 200.00 (173.75, 261.25) | 0.026 |
| Postoperative hospital stay, (median [IQR]), day) | 11.00 (9.00, 13.00) | 11.00 (9.00, 13.00) | 10.00 (9.00, 13.00) | 0.402 |
| **CT measurement parameters** | | | | |
| Tumor size, (median [IQR]), mm) | 35.00 (30.00, 50.00) | 35.00 (27.25, 45.00) | 40.00 (30.00, 60.00) | 0.027 |
| Distance from the tumor to anus, (median [IQR]), cm) | 9.55 (5.43, 11.10) | 9.55 (5.32, 11.07) | 9.55 (6.45, 11.10) | 0.444 |
| SMI, （mean [SD], cm/kg2) | 43.66 ± 8.10 | 44.40 ± 7.59 | 40.93 ± 9.34 | 0.016 |
| SAI, （mean [SD], cm/kg2) | 47.76 ± 21.76 | 52.50 ± 20.67 | 30.46 ± 16.30 | <.001 |
| VAI, （mean [SD], cm/kg2) | 40.72 ± 22.23 | 46.81 ± 20.61 | 18.49 ± 10.85 | <.001 |
| SMD,（mean [SD], U) | 32.67 ± 7.17 | 31.93 ± 6.95 | 35.36 ± 7.44 | 0.007 |
| SAD, （mean [SD], U) | -97.34 ± 9.03 | -99.51 ± 6.07 | -89.43 ± 12.97 | <.001 |
| VAD, （mean [SD], U) | -94.08 ± 8.36 | -96.01 ± 7.25 | -87.00 ± 8.38 | <.001 |
| VSR, (median [IQR]) | 0.79 (0.56, 1.13) | 0.93 (0.62, 1.22) | 0.58 (0.41, 0.74) | <.001 |
| Blood Laboratory indicators |  |  |  |  |
| AAPR, （mean [SD]) | 0.54 ± 0.16 | 0.53 ± 0.15 | 0.54 ± 0.20 | 0.735 |
| IBI, (median [IQR]) | 12.10 (9.43, 17.50) | 11.79 (9.28, 15.47) | 16.27 (10.57, 21.31) | 0.014 |
| PNI, (median [IQR]) | 49.90 (46.23, 52.38) | 50.05 (46.50, 52.68) | 49.70 (45.25, 51.35) | 0.288 |
| CEA, (median [IQR], ng/L) | 3.70 (2.51, 6.54) | 3.61 (2.50, 5.60) | 4.81 (2.76, 10.12) | 0.127 |
| **Pathological** | | | | |
| TNM stage, n(%) |  |  |  | 0.033 |
| I/II | 128 (68.82) | 106 (72.60) | 22 (55.00) |  |
| III | 58 (31.18) | 40 (27.40) | 18 (45.00) |  |
| Nerve invasion, n(%) |  |  |  | 0.14 |
| No | 146 (78.49) | 118 (80.82) | 28 (70.00) |  |
| Yes | 40 (21.51) | 28 (19.18) | 12 (30.00) |  |
| Vascular invasion, n(%) |  |  |  | 0.215 |
| No | 152 (81.72) | 122 (83.56) | 30 (75.00) |  |
| Yes | 34 (18.28) | 24 (16.44) | 10 (25.00) |  |
| LNR, (median [IQR]) | 0.00 (0.00, 0.13) | 0.00 (0.00, 0.14) | 0.00 (0.00, 0.12) | 0.87 |

TNM, Tumor-Node-Metastasis(The 8th edition of the American Joint Committee on Cancer (AJCC) staging system); SMI, skeletal muscle index; SAI, subcutaneous adipose tissue index; VAI, visceral adipose tissue index; SMD, skeletal muscle density; SAD, subcutaneous adipose tissue density; VAD, visceral adipose tissue density; VSR, visceral-to-subcutaneous adipose tissue area ratio; AAPR, albumin-to-alkaline phosphatase ratio; IBI, inflammatory burden index; PNI, prognostic nutritional index; CEA, carcinoembryonic antigen.

**Supplementary Table S2 Evaluate the predictive performance of seven ML models in the trainig cohort.**

| **Indicator** | **Model** | | | | | | |
| --- | --- | --- | --- | --- | --- | --- | --- |
|  | **XGBoost** | **DT** | **SVM** | **RF** | **LightGBM** | **LR** | **KNN** |
| AUROC | 0.922 | 0.925 | 0.894 | 0.902 | 0.935 | 0.949 | 0.924 |
| Accuracy | 0.785 | 0.908 | 0.885 | 0.900 | 0.869 | 0.885 | 0.754 |
| Sensitivity | 0.735 | 0.75 | 0.902 | 0.941 | 0.863 | 0.863 | 0.686 |
| Specificity | 0.964 | 0.951 | 0.821 | 0.750 | 0.893 | 0.964 | 1 |
| PPV | 0.987 | 0.808 | 0.948 | 0.932 | 0.967 | 0.989 | 1 |
| NPV | 0.500 | 0.933 | 0.697 | 0.778 | 0.641 | 0.659 | 0.467 |
| Balance Accuracy | 0.850 | 0.850 | 0.862 | 0.846 | 0.878 | 0.914 | 0.843 |
| Precision | 0.987 | 0.808 | 0.948 | 0.932 | 0.967 | 0.989 | 1 |
| Recall | 0.735 | 0.750 | 0.902 | 0.941 | 0.863 | 0.863 | 0.686 |
| F-Score | 0.843 | 0.778 | 0.925 | 0.937 | 0.912 | 0.921 | 0.814 |

DT, Decision Tree; KNN, K-Nearest Neighbors; LightGBM, Light Gradient Boosting Machine; LR, Logistic Regression; RF, Random Forest; SVM, Support Vector Machine; XGBoost, Extreme Gradient Boosting; PPV, Positive Predictive Value; NPV, Negative Predictive Value.

**Supplementary Table S3 Compare the AUROC differences of the seven models using the DeLong test in the training cohort**

| **Models** | **XGBoost** | **RF** | **LR** | **SVM** | **LightGBM** | **DT** |
| --- | --- | --- | --- | --- | --- | --- |
| **RF** | 0.524 |  |  |  |  |  |
| **LR** | 0.345 | 0.592 |  |  |  |  |
| **SVM** | 0.669 | 0.963 | 0.502 |  |  |  |
| **LightGBM** | 0.691 | 0.688 | 0.482 | 0.85 |  |  |
| **DT** | 0.829 | 0.703 | 0.375 | 0.736 | 0.963 |  |
| **KNN** | 0.024 | 0.029 | 0.121 | 0.002 | 0.038 | 0.063 |

DT, Decision Tree; KNN, K-Nearest Neighbors; LightGBM, Light Gradient Boosting Machine; LR, Logistic Regression; RF, Random Forest; SVM, Support Vector Machine; XGBoost, Extreme Gradient Boosting.

**Supplementary Figure S1 Schematic diagram at the level of the L4/5 spine in the used patient using Slice-O-Matic software.**

**
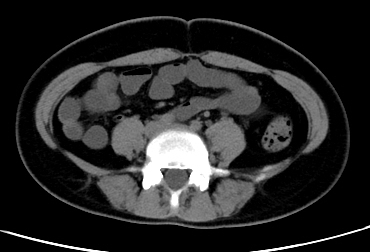

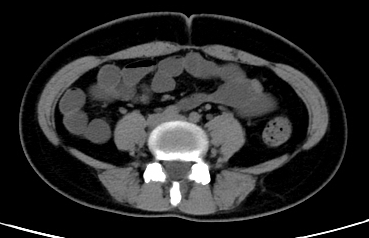
**

**
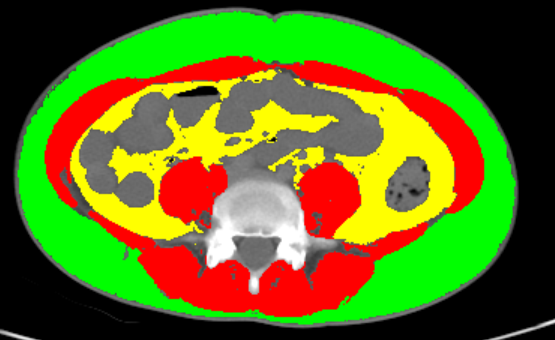

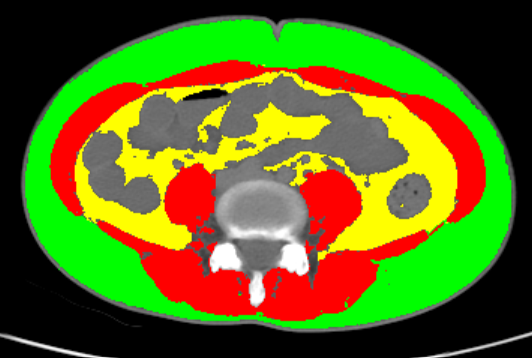
**

Consecutive CT scans and segmentation diagrams of the L4/L5 intervertebral space of a 42-year-old female patient, illustrating subcutaneous adipose tissue (green), visceral adipose tissue (yellow), and skeletal muscle tissue (red). The average values of tissue areas and densities depicted in the two CT scans are as follows: subcutaneous adipose tissue area of 121.5 cm2, visceral adipose tissue area of 63.145 cm2, skeletal muscle tissue area of 88.6 cm2, SAD of -109.1 (U), VAD of -101.15 (U), and SMD of 37.565 (U).

**Supplementary Figure S2 The calibration curves for the seven models.**

**
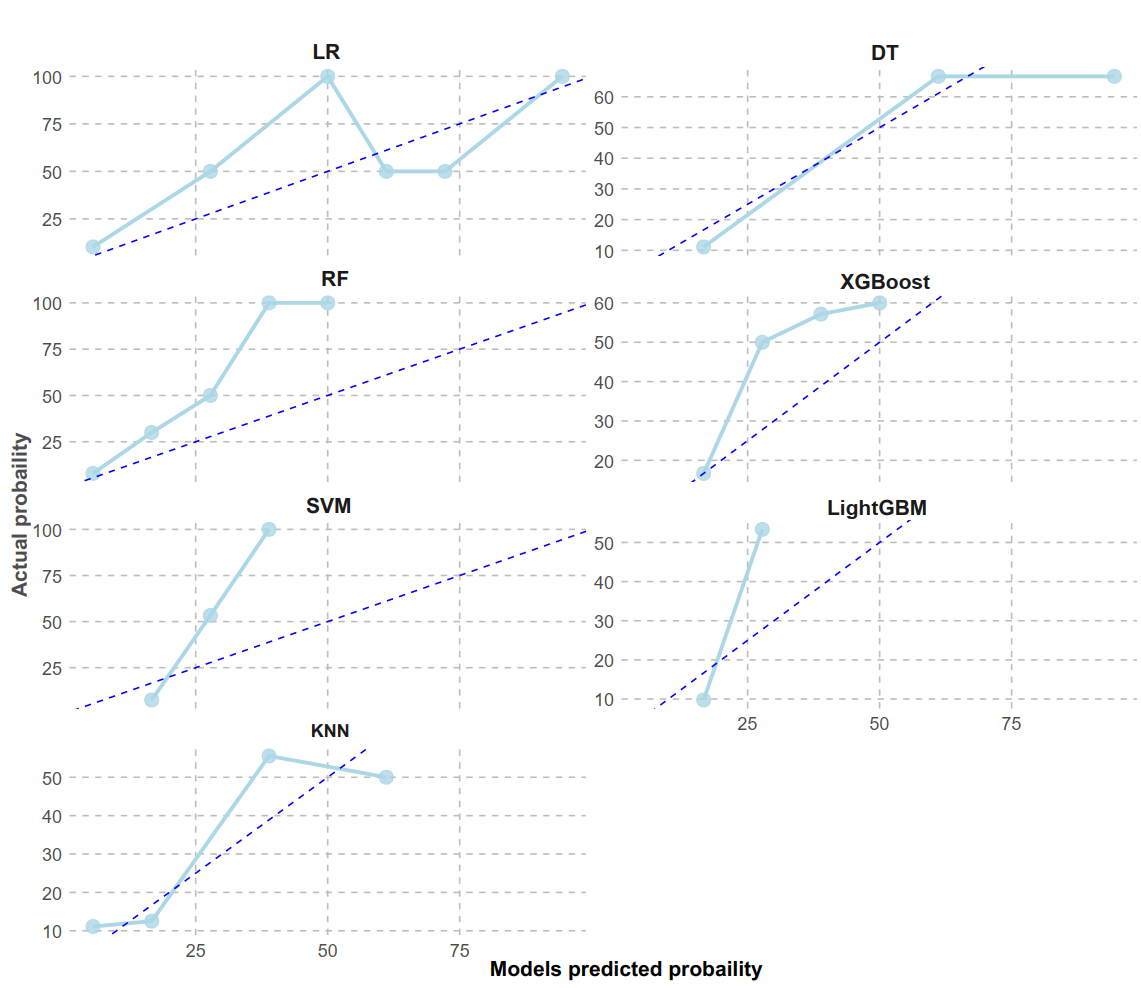
**

DT, Decision Tree; KNN, K-Nearest Neighbors; LightGBM, Light Gradient Boosting Machine; LR, Logistic Regression; RF, Random Forest; SVM, Support Vector Machine; XGBoost, Extreme Gradient Boosting.

**Supplementary Figure S3 The clinical impact curves for the seven models.**

**
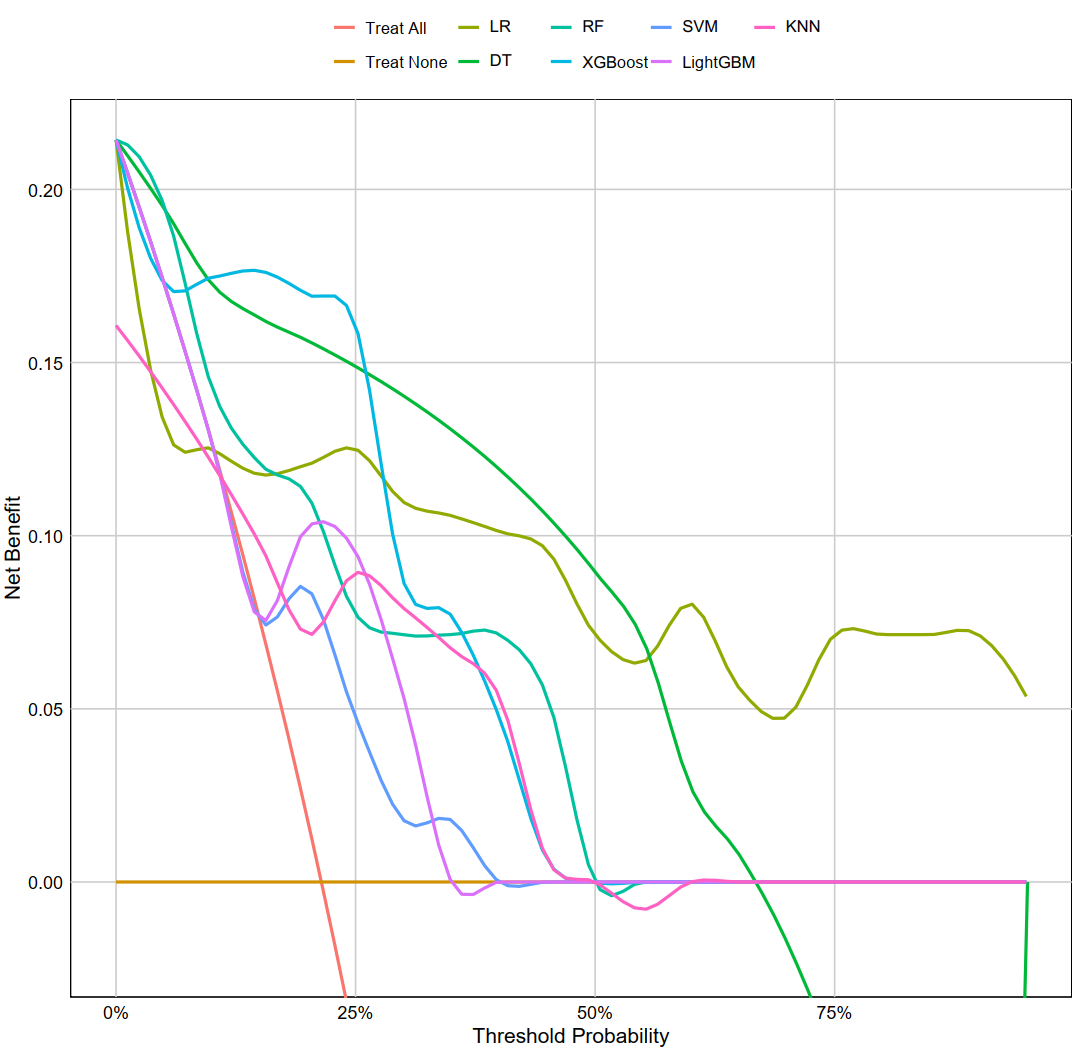
**
